# Editorial: Break the stigma: autism. The future of research on autism stigma - towards multilevel, contextual & global understanding

**DOI:** 10.3389/fpsyt.2024.1504429

**Published:** 2024-11-06

**Authors:** Ava N. Gurba, Morgan L. McNair, April Hargreaves, Nichole E. Scheerer, Catalina Sau Man Ng, Matthew D. Lerner

**Affiliations:** ^1^ AJ Drexel Autism Institute, Drexel University, Philadelphia, PA, United States; ^2^ Department of Psychology, Stony Brook University, Stony Brook, NY, United States; ^3^ Department of Psychology, National College of Ireland, Dublin, Ireland; ^4^ Department of Psychology, Wilfrid Laurier University, Waterloo, ON, Canada; ^5^ Department of Early Childhood Education, The Education University of Hong Kong, Hong Kong, Hong Kong SAR, China; ^6^ Department of Clinical, Educational and Health Psychology, University College London, London, United Kingdom

**Keywords:** autism, stigma, prejudice & discrimination, neurodiversity, mental health, neurodiversity affirming practice, intersectionality

Approaches to understanding and supporting autistic people in the 21st century have shifted from individual-level, often negatively valanced views, to an embrace of the fact that autistic people - like all people - exist in interaction with their context. Additionally, modern approaches have embraced neurodiversity, the idea that all people, regardless of neurocognitive abilities, have value, and highlights ways an individual is shaped in dialogue with their environment (1–4). However, the world is not built with neurodivergence in mind, and when individuals struggle, these difficulties are often pathologized - and stigmatized - across levels of societal experience: public, interpersonal, and internal (Bottema-Beutel et al.).

Historically, public portrayals of autism have been either minimal, caricatured, and/or stereotyped. Recently, there has been a shift, particularly in media, with an increase in portrayals that either offer a positive or more dimensional representation of autistic experiences (5–7). That said, the overall landscape remains far from balanced. For example, recent work shows that artificial intelligence-generated views and images of autism predominantly maintain a negative, stigmatized depiction of autism (8, 9), offering a data-driven mirror into public perceptions. It is notable, however, that some of the most effective instances of authentic portrayals have arisen from a *participatory* approach, whereby autistic people are either directly involved in the development of the characterization of a person’s experience, and/or are the individuals representing (or represented in) those portrayals (10–12). Research has started to identify the impact of these portrayals on stigma, with initial evidence suggesting both increases and decreases in negative views (and self-views) of autistic people via these representations (7, 12–15). Thus, ongoing work should seek to examine the impact of different approaches to inclusion on the stigma that autistic people experience.

Stigma can also manifest in interpersonal relationships (e.g., family, classmates, work colleagues, other peers), in ways that can ultimately become enacted as prejudice. Thus, there is an urgent need to understand and address the ways in which interpersonal stigma is established and maintained (Marion et al.). Many established interventions designed to address stigma in social settings (e.g., peer relations) have used approaches that do little to address this outcome, and, at times, make it worse, leading to increased interpersonal victimization and risk of direct harm (16, 17). They often target the social behaviors of *autistic* people, with the aim of normalizing their behaviors to be more tolerable to non-autistic peers. This effect can then metastasize into systemic prejudice, wherein systems (e.g., employment, legal involvement) (18, 19) can themselves reflect these same expectations and values. Notably, recent work has begun to take a different path, seeking to provide psychoeducation to peers, colleagues, and the public about neurodiversity and the range of lived experiences these encompass (20–23). Intervention approaches have also started to take a more performance-based approach, aiming to advance each person’s strengths and encouraging *choice* rather than behavioral normalization (24, 25). However, these approaches are often small-scale and consider only a subset of contexts, only beginning to scratch the surface of the need in this domain.

The culmination of these contextual forms of stigma for autistic people is that they can become internalized, and deeply impact how individuals view themselves. While the impact of internalized stigma is well-documented in other minoritized communities (26, 27), the consideration or prioritization of *any* subjective experiences *at all* has, until recently, been largely ignored in autism research. Several areas where work has started to take root include the impact on autistic identity formation and integration (28, 29), masking or passing as non-autistic (30, 31), minority stress (28, 32), internalized ableism (33), and distress experiences (34). However, other outcome domains, including shame, advocacy and self-advocacy, autistic community (35), and the perceptions of and prejudice towards other autistic people (i.e., lateral ableism) (36–38), have barely begun to be examined. Notably, these impacts represent areas that have long been advocated as an area of focus by the autism community, and many of the efforts to address autism stigma to date have largely been advanced by the efforts of autistic individuals, advocates, and scholars.

With the rise of the neurodiversity movement, many autistic people have realized a distinct marginalized identity, characterized by diverse strengths and challenges; this movement has also helped to identify acceptable unique supports to more inclusively address the needs of autistic individuals (17, 18). The autism research field, then, has finally started to recognize how the experience and impact of stigma and prejudice on autistic people can manifest in ways similar to (32) - and distinct from - that of other minority groups. This highlights the importance of learning from the diverse experiences of autistic people within and across cultures, communities, identities, and backgrounds, which could reinforce *or* bolster against adverse experiences of stigma and prejudice (28, 36, 39–43, Yoon et al.). More broadly, these developments highlight the importance of taking a capacious approach to addressing stigma and prejudice in this field, as represented by this Research Topic.

This work, then, can spur deeper investigations that can advance understanding and – ultimately – actionable findings that can continue to change the ways autistic people are viewed and supported in the community. Finally, we note that nearly all of these changes to date have been driven by the advocacy of autistic people at each of these levels, highlighting that, as ever, the best bulwarks against the effects of stigma remain the unified efforts of a marginalized community themselves. Therefore, it is our hope that this Research Topic can act as a catalyst and a call–to–arms for more dedicated research by both non–autistic and autistic researchers, clinicians, and other professionals alike, aiming to identify and address autism stigma and its impacts on autistic people and society around the globe, particularly in ways that continue to center the experience and contribution of autistic people, targeted efforts to address the breadth of stigma and prejudice experiences of autistic people of all backgrounds, and efforts to address existing stigma and prejudice within the autistic community, in efforts to reduce intra–community harms and increase collective action, thereby promoting a more equitable, inclusive world for all autistic people.
